# Complete and Early Vitrectomy for Sterile Endophthalmitis After Bevacizumab: A Case Series

**DOI:** 10.7759/cureus.93996

**Published:** 2025-10-07

**Authors:** Agnieszka Kudasiewicz-Kardaszewska, Malgorzata A Ozimek, Aleksandra Kardaszewska, Piotr Kardaszewski, Natalia Kapturska, Kinga Jamontt, Karolina Boninska, Slawomir Cisiecki

**Affiliations:** 1 Ophthalmology, Professor Zagórski Eye Surgery Centre, OCHO Medical Group, Nowy Sącz, POL; 2 Dentistry, Division of Medical Faculty, Medical University of Lublin, Lublin, POL; 3 General Medicine, Samodzielny Publiczny ZOZ MSWiA w Lublinie, Lublin, POL; 4 Ophthalmology, Miejskie Centrum Medyczne im. dr. Karola Jonschera w Łodzi, Łódź, POL

**Keywords:** bevacizumab, ceve protocol, intravitreal injection, sterile endophthalmitis, vitrectomy

## Abstract

Intravitreal anti-vascular endothelial growth factor injections are widely used for retinal disorders but may occasionally lead to sterile endophthalmitis, a condition that can be challenging to distinguish from infectious endophthalmitis. We describe a series of five patients who developed acute intraocular inflammation within 24-48 hours of receiving bevacizumab injections. These patients presented with decreased visual acuity and vitreous haze but reported minimal or no pain. All patients were managed urgently with pars plana vitrectomy according to the complete and early vitrectomy for endophthalmitis (CEVE) protocol, which included intraoperative antibiotic infusion and microbiological sampling. Cultures from the vitreous samples and the bevacizumab vial were negative. Following early vitrectomy, inflammation resolved in all patients, and visual acuity returned to baseline within 30 days. No postoperative complications were observed. None of the 38 additional patients who received injections from the same vial developed endophthalmitis. These findings support an immune-mediated, noninfectious mechanism for these cases and suggest that early vitrectomy within the CEVE framework promotes rapid resolution and favorable visual recovery. Continued vigilance and further research are warranted to refine management strategies for sterile endophthalmitis.

## Introduction

Anti-vascular endothelial growth factor (anti-VEGF) injections are widely used for retinal diseases such as age-related macular degeneration (AMD), diabetic macular edema (DME), and cystoid macular edema (CME) associated with retinal vein occlusion (RVO) [1,2]. Although generally safe, they may rarely cause severe complications, including infectious endophthalmitis (reported in 0.02-0.14% of cases) and sterile endophthalmitis (0.005-4.4%) [3-5].

Sterile endophthalmitis is an intraocular inflammatory reaction without infection, typically presenting with reduced vision, minimal pain, and vitreous haze [6-10]. Its pathogenesis has been associated with immune mechanisms or drug-related factors such as preservatives, endotoxins, or protein aggregates [7,9,10]. Bevacizumab, in particular, has been linked to clusters of sterile inflammation, often attributed to repackaging and storage issues [8,10-12]. Additionally, silicone oil released from syringes can induce immunogenic protein aggregates. Factors such as syringe agitation, freeze-thaw cycles, shipping, and improper storage prior to injection may increase silicone oil release [10]. Differentiating sterile from infectious endophthalmitis at presentation remains challenging, complicating management decisions [9,10].

The complete and early vitrectomy for endophthalmitis (CEVE) protocol advocates immediate and complete vitrectomy, which facilitates rapid clearance of inflammatory material, enhances intraocular drug penetration, and reduces the risk of retinal damage [13-16]. This approach contrasts with the conventional “tap-and-inject” strategy, in which vitreous sampling and intravitreal antibiotics are performed first, and vitrectomy is delayed or reserved for severe cases [14]. The CEVE protocol promotes immediate surgical intervention and represents a promising option when distinguishing between sterile and infectious endophthalmitis is difficult. Studies have demonstrated improved outcomes and reduced complications with this proactive approach [15-17].

While CEVE has shown encouraging results in infectious endophthalmitis, its role in sterile cases is not well established [16]. Here, we present a case series of presumed sterile endophthalmitis following bevacizumab (Avastin; intravitreal dose 1.25 mg/0.05 ml; Roche, Basel, Switzerland) injection, successfully managed with CEVE. This report addresses a gap in the current literature on the surgical management of noninfectious, post-injection inflammation. Our findings suggest that CEVE may be a preferable strategy when the distinction between infectious and sterile endophthalmitis is uncertain.

## Case presentation

We retrospectively reviewed electronic medical records of patients who developed acute intraocular inflammation within 24-48 hours following intravitreal injection of bevacizumab (Avastin, Roche; 1.25 mg/0.05 ml administered via pars plana) at Professor Zagórski Eye Surgery Centre (Nowy Sącz, OCHO Medical Group, Poland).

The inclusion criteria were (1) a recent intravitreal bevacizumab injection performed under standard sterile operating room (OR) conditions; (2) new-onset decreased visual acuity with vitreous haze and minimal or no ocular pain; and (3) availability of complete follow-up data.

All injections were performed in the OR under full sterile conditions on August 21, 2024. Personnel wore standard OR attire, including masks covering the nose and mouth. The operator and scrub nurse additionally wore sterile gowns and gloves. Each patient entered the OR wearing single-use attire, a cap, and a mask covering the nose and mouth. A “no talking” policy was also standard.

Prior to injection, proxymetacaine (Alcaine, Alcon Laboratories, Puurs, Belgium) was instilled into the eye after the application of 5% povidone iodine (PI). In the OR, the skin of the eyelids, eyelashes, and periocular area was disinfected using 10% PI for 30 seconds. A sterile drape with foil and a speculum was then applied, followed by additional instillation of Alcaine and 5% PI for another 30 seconds.

The injection was performed under a microscope via pars plana, 3.5 mm from the limbus in pseudophakic eyes and 4 mm from the limbus in phakic eyes. Bevacizumab was withdrawn from the vial using an antimicrobial filter into an integrated needle syringe immediately before injection. The vial was stored at room temperature under a sterile drape. After the injection, 5% PI and ofloxacin ointment (Floral, Dr. Gerhard Mann Chem.-Pharm. Fabrik GmbH, Berlin, Germany) were applied to the ocular surface. No post-procedural medications, including antibiotics, were prescribed [5,10].

On the injection day, 43 eyes received bevacizumab; only five developed sterile endophthalmitis.

Case 1

**Figure 1:**
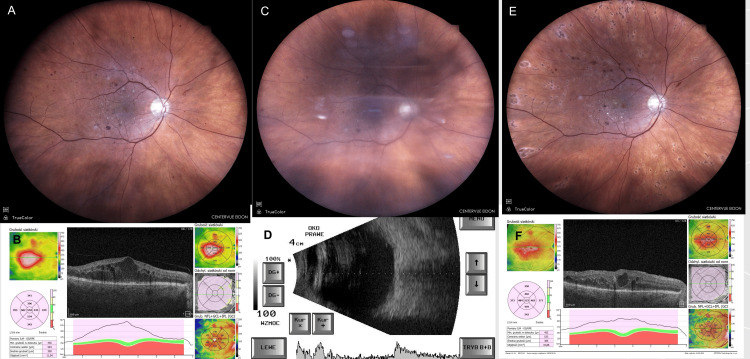
Case 1: patient treated for DME who developed endophthalmitis after the sixth bevacizumab injection. (A) Before inflammation: DME with maculopathy, fundus photo (taken on the day of injection). (B) Before inflammation: DME, OCT image (taken on the day of injection). (C) Sterile endophthalmitis: retina barely visible, fundus photo (taken 24 hours after injection). (D) Sterile endophthalmitis: B-scan ultrasound (taken 24 hours after injection). (E) One month after CEVE: fundus photo (taken 30 days after CEVE). (F) Two months after CEVE: residual DME on OCT (taken 60 days after CEVE). Images taken at OCHO Nowy Sącz. Fundus photographs: iCare EIDON FA, iCare, Vantaa, Finland. OCT: Revo NX80 SOCT, Optopol Technology, Zawiercie, Poland. USG: Echoson, Echo-Son S.A., Warsaw, Poland. The OCT image during inflammation (corresponding to Figure 1C) could not be obtained due to vitreous inflammatory opacities; therefore, a B-scan ultrasound was included instead. Timeline: injection (August 21, 2024) → inflammation (August 22, 2024) → CEVE (August 22, 2024) → postoperative follow-up visits (seven and 30 days postoperatively). CEVE, complete and early vitrectomy for endophthalmitis; DME, diabetic macular edema; OCT, optical coherence tomography; RE, right eye

A 64-year-old male treated for DME presented for consultation the day after his injection. The patient had received an intravitreal bevacizumab injection to the right eye (RE), marking his sixth injection. He reported rapid visual decline accompanied by mild pain 24 hours post-injection. The attending physician diagnosed subacute endophthalmitis in the RE and immediately notified the vitreoretinal surgeon on duty. The patient was urgently scheduled for vitrectomy following the CEVE protocol. During surgery, a vitreous sample was collected for culture (Figure 1).

The next day, in the late morning hours, four more patients presented with similar symptoms, including rapid vision deterioration, mild or no pain, and an inflammatory reaction in the vitreous body (mid-vitreous cavity).

Case 2

**Figure 2:**
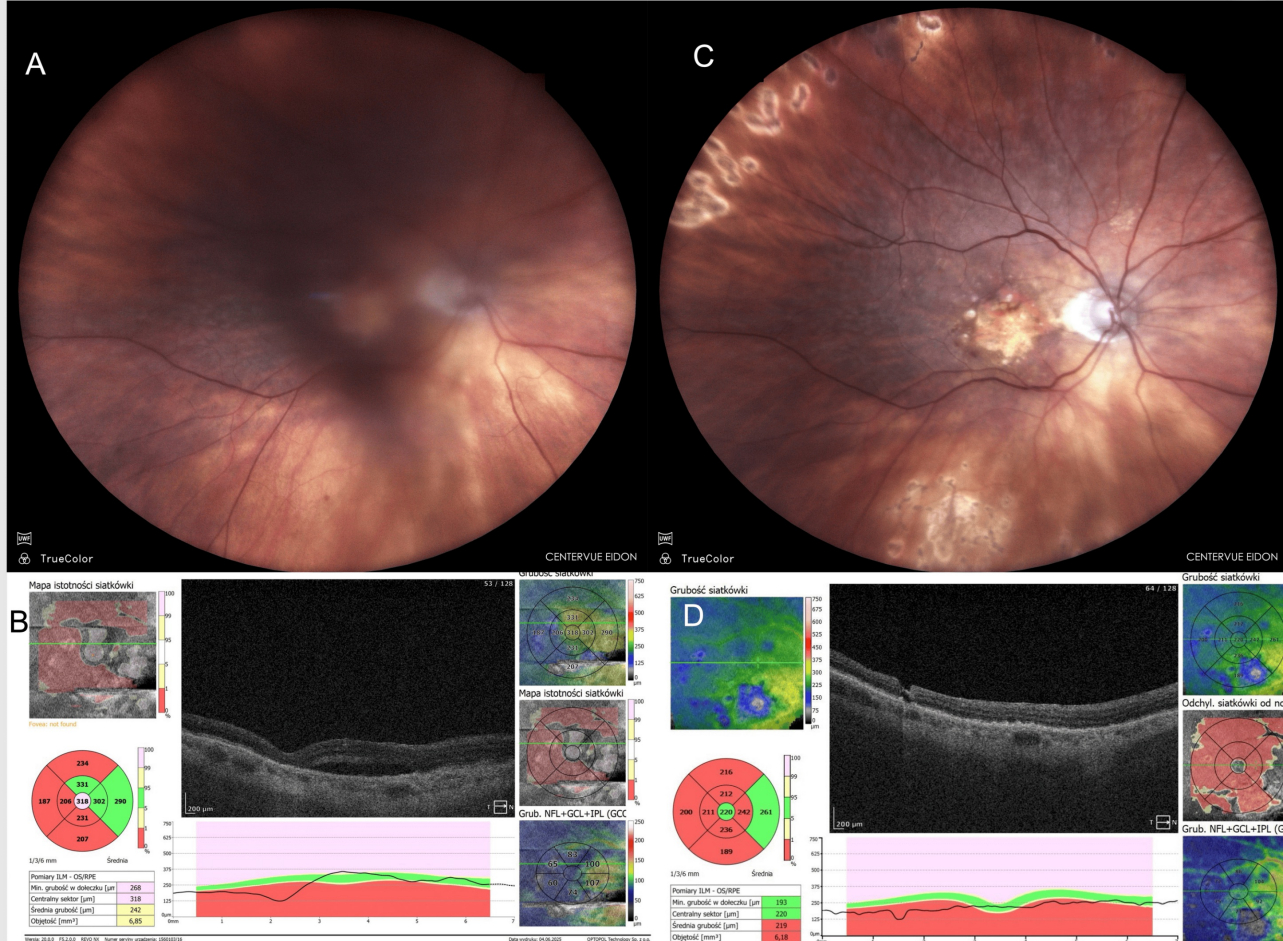
Case 2: 86-year-old female. Vitritis developed after the 13th intravitreal bevacizumab injection for AMD in the RE. (A) Fundus photograph showing the central retina barely visible due to vitritis (taken 48 hours after injection). (B) OCT image taken before CEVE showing poor visibility due to vitritis (taken 48 hours after injection). (C) Fundus photograph one month after CEVE (taken 30 days postoperatively). (D) OCT image one month after CEVE (taken 30 days postoperatively). Images taken at OCHO Nowy Sącz. Fundus photographs: iCare EIDON FA, iCare, Vantaa, Finland. OCT: Revo NX80 SOCT, Optopol Technology, Zawiercie, Poland. Timeline: Injection (August 21, 2024) → Inflammation (August 23, 2024) → CEVE (August 23, 2024) → Postoperative follow-up visits at seven and 30 days postoperatively. AMD, age-related macular degeneration; CEVE, complete and early vitrectomy for endophthalmitis; OCT, optical coherence tomography; RE, right eye

An 86-year-old female reported decreased visual acuity and “fogging” in her RE, with almost no pain, 48 hours after an intravitreal injection. Bevacizumab was administered for AMD, and this was her 13th injection in the same eye (Figure 2).

Case 3

**Figure 3:**
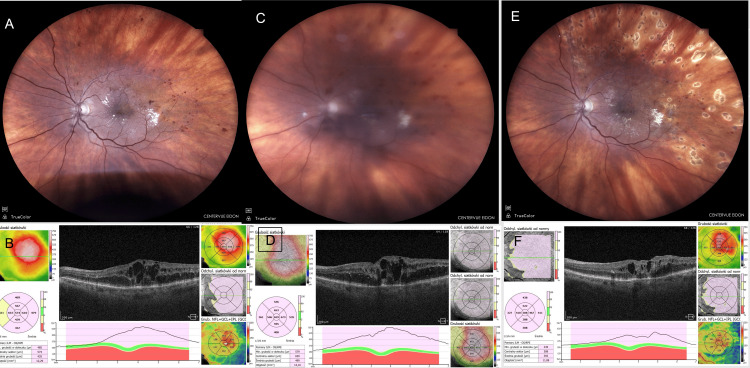
Case 3: 82-year-old female. Sterile endophthalmitis during bevacizumab injections for CME secondary to BRVO in the LE. (A) Fundus photograph showing CME due to BRVO before inflammation (taken before injection). (B) OCT image showing CME (taken before injection). (C) Fundus photograph with noticeable vitritis obscuring the macular region (taken before CEVE, 48 hours after injection). (D) OCT image during inflammation showing increased CME; image quality is poor due to vitritis (taken before CEVE). (E) Postoperative fundus photograph (taken seven days postoperatively). (F) Postoperative OCT image showing decreased but persistent CME (taken seven days postoperatively). Images taken at OCHO Nowy Sącz. Fundus photographs: iCare EIDON FA, iCare, Vantaa, Finland. OCT: Revo NX80 SOCT, Optopol Technology, Zawiercie, Poland. Timeline: Injection (August 21, 2024) → Inflammation (August 23, 2024) → CEVE (August 23, 2024) → Postoperative follow-up visits at seven and 30 days postoperatively. BRVO, branch retinal vein occlusion; CEVE, complete and early vitrectomy for endophthalmitis; CME, cystoid macular edema; LE, left eye; OCT, optical coherence tomography

An 82-year-old female developed similar symptoms and signs in her left eye (LE) 36 hours after injection. Bevacizumab was administered for macular edema secondary to branch RVO (BRVO). This was also a subsequent injection in the same eye (Figure 3).

Case 4

**Figure 4:**
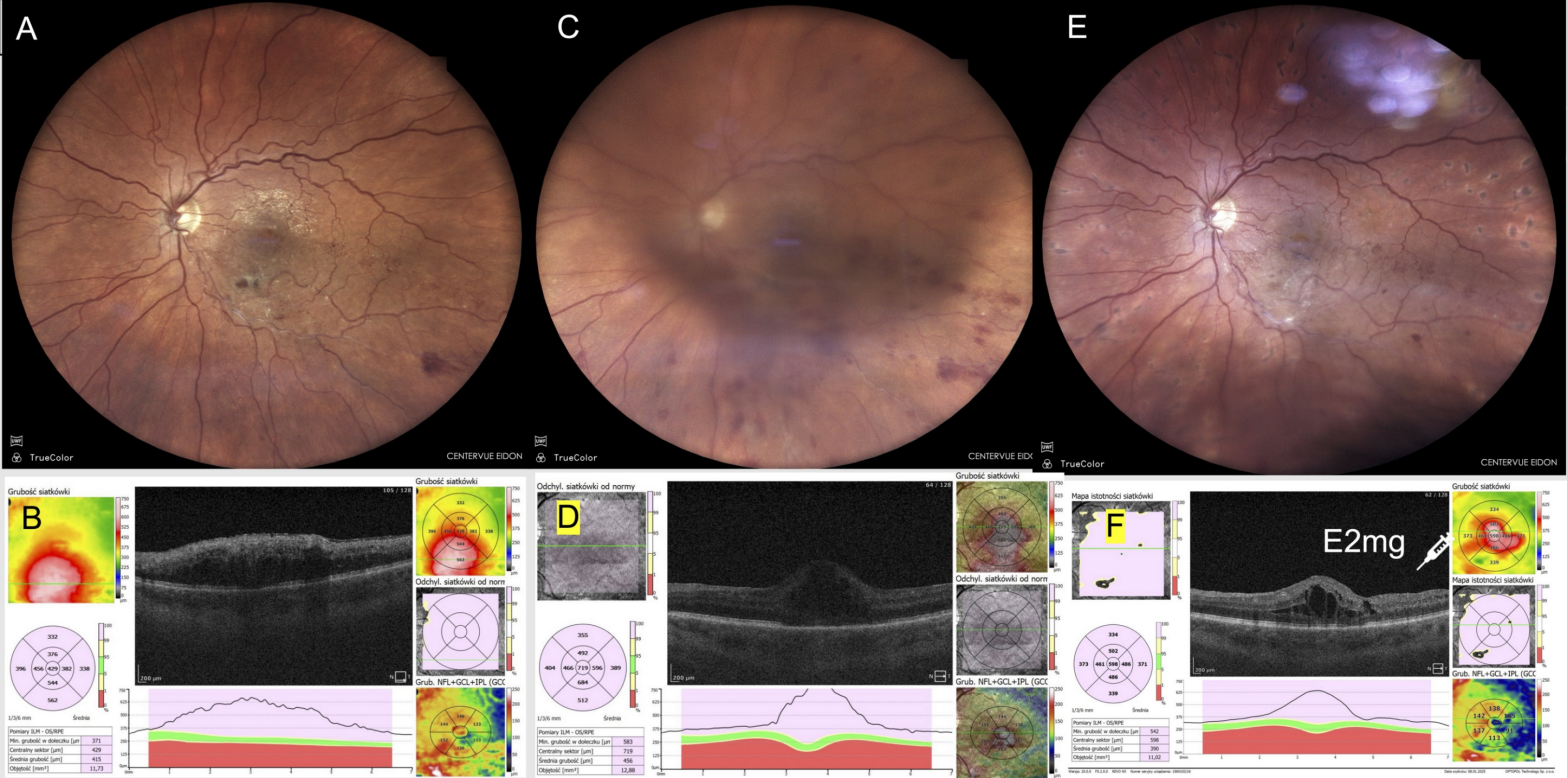
Case 3: 58-year-old woman with bevacizumab due to CME in the course of BRVO in the LE, 1st injection to the LE. (A) Fundus photograph taken before injection. (B) OCT image showing CME before injection. (C) Fundus photograph showing sterile endophthalmitis with a hazy vitreous appearance (taken before CEVE, 48 hours after injection). (D) OCT image during endophthalmitis showing CME; image appears hazy due to vitreous opacities (taken before CEVE, 48 hours after injection). (E) Fundus photograph taken 30 days postoperatively. (F) OCT image of the LE 30 days postoperatively. Anti-VEGF treatment with aflibercept 2 mg (Eylea 2 mg, Bayer - E2 mg) was continued due to CME. Images taken at OCHO Nowy Sącz. Fundus photographs: iCare EIDON FA, iCare, Vantaa, Finland. OCT: Revo NX80 SOCT, Optopol Technology, Zawiercie, Poland. Timeline: Injection (August 21, 2024) → Inflammation (August 23, 2024) → CEVE (August 23, 2024) → Postoperative follow-up visits at seven and 30 days postoperatively. anti-VEGF, anti-vascular endothelial growth factor; BRVO, branch retinal vein occlusion; CEVE, complete and early vitrectomy for endophthalmitis; CME, cystoid macular edema; LE, left eye; OCT, optical coherence tomography

A 58-year-old female reported decreased visual acuity, mild pain in her LE, and vitritis within 48 hours after injection. Bevacizumab was administered for macular edema due to BRVO. This was the first dose of bevacizumab administered to this eye (Figure 4).

Case 5

**Figure 5:**
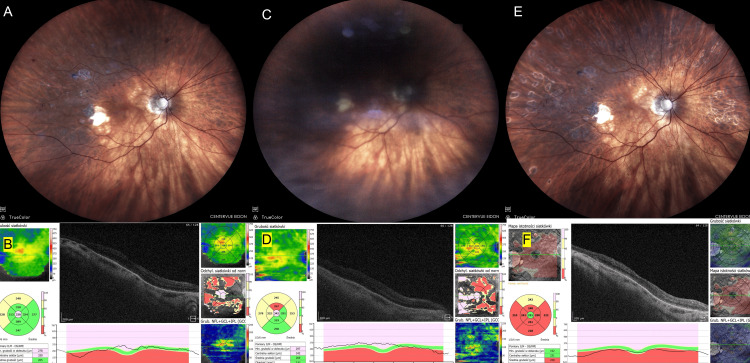
Case 5: 72-year-old male with CME secondary to BRVO. (A) Fundus photograph taken before the bevacizumab injection. (B) OCT image taken before injection. (C) Fundus photograph showing inflammation with vitritis obscuring the macula (taken before CEVE, 48 hours after injection). (D) OCT image during inflammation showing a blurry macular appearance due to vitreous haze (taken before CEVE, 48 hours after injection). (E) Fundus photograph one month after CEVE (taken 30 days postoperatively). (F) OCT image one month after CEVE showing resolution of CME (taken 30 days postoperatively). Images taken at OCHO Nowy Sącz. Fundus photographs: iCare EIDON FA, iCare, Vantaa, Finland. OCT: Revo NX80 SOCT, Optopol Technology, Zawiercie, Poland. Timeline: Injection (August 21, 2024) → Inflammation (August 23, 2024) → CEVE (August 23, 2024) → Postoperative follow-up visits at seven and 30 days postoperatively. BRVO, branch retinal vein occlusion; CEVE, complete and early vitrectomy for endophthalmitis; CME, cystoid macular edema; OCT, optical coherence tomography

A 72-year-old male developed vitritis without pain and worsening visual acuity in his RE 48 hours after injection. Bevacizumab was administered for macular edema secondary to BRVO (Figure 5).

CEVE protocol for the surgical procedure

All affected patients underwent pars plana vitrectomy following the CEVE protocol, with the key steps outlined below. After trocar insertion, samples from the vitreous cavity and anterior chamber were collected for microbiological testing. During vitrectomy, a broad-spectrum antibiotic (vancomycin 0.2 mg/ml in 500 ml Ringer’s solution; Vancomycin-MIP, MIP Pharma Ltd., Gdańsk, Poland) was administered via the infusion fluid [17]. Intraoperative anterior chamber lavage was performed using another broad-spectrum antibiotic, moxifloxacin (500 µg/0.02 ml, Vigamox, Novartis Warsaw, Poland) [12,13].

All inflammatory debris in the vitreous cavity was carefully removed, ensuring posterior vitreous detachment (PVD) to thoroughly clean the retinal surface. In all cases, internal limiting membrane (ILM) peeling was performed using green dye (indocyanine green - ICG, soluble in 5% glucose; Verdye, Diagnostic Green Ltd, Athlone, Ireland) to prevent potential central proliferative vitreoretinopathy (PVR) reaction [13].

At the end of the procedure, moxifloxacin (500 µg/0.1 ml) and vancomycin (1 mg/0.1 ml) were injected into the anterior and vitreous chambers, respectively [16]. Sulphur hexafluoride (SF₆) gas at a 20% concentration was then introduced into the eye. Postoperatively, all patients received topical levofloxacin + dexamethasone (Ducressa, Santen Oy, Tampere, Finland) five times daily for seven days, followed by tapering dexamethasone monotherapy (0.1% Dexamethasone WZF, Zakłady Farmaceutyczne POLPHARMA S.A., Starogard Gdański, Poland) over three weeks [17].

Fundus photography (iCare EIDON FA, iCare, Vantaa, Finland), OCT (Revo NX80 SOCT, Poland), and ultrasonography (Echoson, Echo-Son S.A., Puławy, Poland) were used for documentation before surgery and during follow-up visits.

Microbiological cultures were performed from vitreous samples, anterior chamber fluid, and the residual bevacizumab vial using blood agar and chocolate agar (for general recovery and Gram-positive bacteria) and MacConkey agar (for Gram-negative rods). Cultures were incubated for 48 hours at 37°C. PCR testing was unavailable.

Internal review revealed that the five affected patients had received their injections at different times throughout the day. Although the same bevacizumab vial was used, there were no consecutive cases. Follow-up teleconsultations with the remaining 38 patients who received injections from the same vial on that day confirmed no additional cases of endophthalmitis.

Culture samples from the five patients, the bevacizumab vial, and the operating room environment (operating table, scrub nurse table, and injection tray) were all negative. The postoperative course was uneventful, and all patients regained their baseline visual acuity within one month. No serious postoperative complications were observed. Table 1 summarizes the patients’ demographic data, diagnoses, and best-corrected visual acuity results.

**Table 1:** Clinical outcomes of sterile endophthalmitis cases managed with the CEVE protocol AMD, age-related macular degeneration; anti-VEGF, anti-vascular endothelial growth factor; BRVO, branch retinal vein occlusion; CEVE, complete and early vitrectomy for endophthalmitis; DME, diabetic macular edema; logMAR, logarithm of the minimum angle of resolution; VA, visual acuity Table credit: Aleksandra Kardaszewska

Case	Age (year)	Eye	Diagnosis (anti-VEGF indication)	VA before CEVE (logMAR)	VA after CEVE (logMAR)	Time to recovery (days)	VA one month after CEVE (logMAR)	Improvement (logMAR)	Complications
1	64	RE	DME	1.40	0.52	18	1.40	0.88	No
2	86	RE	AMD	1.70	1.4	20	1.70	0.70	No
3	82	LE	BRVO	1.40	1.0	22	1.40	0.80	No
4	56	LE	BRVO	1.22	1.22	22	1.22	0.62	No
5	72	RE	BRVO	1.00	1.0	7	1.00	0.30	No
Mean	72	-	-	1.34	1.03	17.8	0.68	0.66	-
Median	72	-	-	1.40	1.0	20	1.40	0.70	-

## Discussion

Sterile endophthalmitis has been documented following intravitreal administration of both anti-VEGF agents and corticosteroids, although its pathophysiology remains incompletely understood [6,7,10,18,19]. Proposed mechanisms include residual impurities, traces of endotoxin, protein aggregation, or antibody-related hypersensitivity reactions [20-26]. Several reports emphasize batch-specific variability, with some bevacizumab lots (Avastin, Roche) demonstrating higher endotoxin levels despite meeting pharmacopeial standards [24-26]. All bevacizumab preparations tested in our series were compliant with pharmacopeial endotoxin limits (Ph. Eur. 2.6.14, harmonized) [26]. However, these standards are based on systemic parenteral use.

The human eye is considerably more sensitive to endotoxin exposure than systemic circulation; thus, preparations considered “compliant” for intravenous administration may still trigger significant intraocular inflammation. This discrepancy may explain why sterile endophthalmitis can occur despite adherence to pharmacopeial quality specifications. Panel experts in retinal surgery have also highlighted aerosolized droplets containing oral contaminants from patients and/or health care providers as a potential infection source. They emphasized the continued importance of PI application and avoiding eyelid contact with the intended injection site and needle [25]. This underscores the need for stricter compounding protocols and endotoxin testing of ophthalmic preparations.

Clinically, sterile inflammation usually presents with moderate visual loss, vitreous haze, and only mild discomfort, but without hypopyon [10,27,28]. In contrast, infectious endophthalmitis typically manifests with severe pain, rapid vision decline, and positive microbiological cultures, although 30-60% of cases remain culture-negative [10,27]. Appendix B shows the possible differentiation between infectious and noninfectious (sterile) endophthalmitis [10]. Advanced molecular diagnostics such as PCR-based 16S rRNA sequencing may further assist in distinguishing infection from sterile inflammation, although this method was not available in our microbiology laboratory [29-33].

In our series, all patients presented within 24-48 hours of injection with mild pain and vitreous haze. Cultures from both eyes and the shared bevacizumab vial were negative, supporting a sterile etiology.

Traditional management of suspected endophthalmitis has relied on intravitreal antibiotics with or without corticosteroids, reserving vitrectomy for severe cases [14,15,31]. However, accumulating evidence suggests that early vitrectomy improves outcomes by clearing inflammatory mediators, enhancing drug penetration, and reducing the risk of complications such as epiretinal membranes, retinal detachment, or PVR [13,15,34,35]. Recent analyses of more than 100 endophthalmitis cases confirm that immediate small-gauge vitrectomy results in visual improvement irrespective of baseline visual acuity [35]. In our series, adherence to the CEVE protocol, including complete vitreous clearance, intraoperative antibiotics, and gas tamponade, resulted in full and rapid recovery without complications.

Comparative data further highlight bevacizumab’s potential risks relative to other anti-VEGF agents. Several studies have found a significantly higher incidence of noninfectious endophthalmitis with bevacizumab (0.081%) compared with ranibizumab (0.005%) [17]. Kiss et al. (2018) reported endophthalmitis rates of 0.100% for aflibercept, 0.056% for bevacizumab, and 0.047% for ranibizumab [11]. Mun et al. confirmed this trend, with incidences per 10,000 injections of 3.64 for bevacizumab, 1.39 for ranibizumab, and 0.76 for aflibercept [18]. These findings support the view that sterile inflammation is uncommon overall but occurs more frequently with bevacizumab, presumably due to repackaging and storage requirements [33].

Our results align with these observations: all five patients developed acute but mild inflammation from the same vial, while no additional cases occurred among 38 other exposed individuals, suggesting a batch-specific reaction. The rapid vision decline justified immediate CEVE rather than the conventional “tap-and-inject” approach, and outcomes were uniformly favorable.

Limitations and future directions

The main limitation of this series is its small sample size (n = 5) and the absence of cytokine or biomarker analyses to confirm immune-mediated pathways [34]. Future research should explore biomarkers such as IL-6 or TNF-α to predict susceptibility to sterile inflammation and evaluate the role of PCR-based diagnostics in ruling out occult infection. Broader adoption of prefilled, single-use bevacizumab syringes is advisable to minimize contamination risk [21,34]. Patient education and early post-injection monitoring remain essential for timely detection and management [11,28].

## Conclusions

Sterile endophthalmitis is an uncommon but significant complication following intravitreal anti-VEGF injections. Early recognition, differentiation from infectious endophthalmitis, and optimal management are crucial to prevent long-term visual impairment. The CEVE protocol offers an effective treatment strategy, as demonstrated in our case series. Immediate vitrectomy for all acute endophthalmitis cases appears to be a proactive and effective approach to this potentially serious condition.

## Appendices

Appendix A 

**Table 2:** Best corrected VA beyond one month AMD, age-related macular degeneration; BRVO, branch retinal vein occlusion; CEVE, complete and early vitrectomy; CME, cystoid macular edema; DME, diabetic macular oedema; LE, left eye; logMAR, logarithm of the minimum angle of resolution; RE, right eye; VA, visual acuity Table credits: Agnieszka Kudasiewicz-Kardaszewska and Aleksandra Kardaszewska

Case	Age (year)	Eye	Diagnosis for anti-VEGF therapy	Baseline VA (before injection)	VA before CEVE (logMAR)	VA one month after CEVE (logMAR)	VA three months after CEVE	Explanation
1	64	RE	DME	0.6	1.40	1.40	0.52	Improvement, but on afibercept 2 mg
2	86	RE	AMD	1.70	1.70	1.70	1.70	Vision stable, dry AMD
3	82	LE	BRVO	0.7	1.40	1.40	0.6	Beyond three months; no data - patient did not appear for the next visit
4	56	LE	BRVO	0.60	1.22	1.22	1.3	Recurrent CME for aflibercept 2 mg
5	72	RE	BRVO	0.6	1.00	1.00	1.0	Recurrent CME for aflibercept 2 mg

Appendix B 

**Table 3:** Clinical differences between sterile and infectious endophthalmitis Table created by Agnieszka Kudasiewicz-Kardaszewska based on literature findings [10,11,18,22]

Feature	Sterile endophthalmitis	Infectious endophthalmitis
Incidence	Rare, reported between ~0.005% and 4.4% of injections	Less common overall, typically 0.02-0.14% of injections
Typical onset	Usually within one to three days, most cases appear in the first 48 hours	Median ~4 days, though onset may range from the same day up to about three to four weeks
Initial visual acuity	Frequently reduced (median ~0.13), but wide range (0.8 to hand motion); about 1 in 10 reach hand motion level	In the majority (>80%), vision is ≤0.05 at presentation
Ocular pain	Uncommon, mild in ~5–10%	Marked pain present in most patients (~70%+)
Hypopyon	Rare (~5%)	Common (seen in >80%)
Conjunctival hyperemia	Occasionally observed (~10%)	Very frequent (>80%)
Resolution time	Generally short, three to five weeks with appropriate management	Variable, dependent on organism and promptness of therapy
Visual outcome	Generally favorable; small minority (~15%) lose more than 0.2 logMAR	Prognosis guarded; permanent visual impairment is common
